# Mechanistic Insights of Qingre Jiedu Recipe Based on Network Pharmacology Approach against Heart Failure

**DOI:** 10.1155/2022/9024394

**Published:** 2022-01-31

**Authors:** Xuan Li, Mingyan Shao, Zhen Liu, Xiaoqian Sun, Lingwen Cui, Xiangning Liu, Gang Wang, Linghui Lu, Yan Wu, Chun Li

**Affiliations:** ^1^School of Chinese Medicine, Beijing University of Chinese Medicine, Beijing 100029, China; ^2^School of Life Sciences, Beijing University of Chinese Medicine, Beijing 100029, China; ^3^Beijing First Hospital of Integrated Chinese and Western Medicine, Beijing 100026, China; ^4^Modern Research Center for Traditional Chinese Medicine, Beijing University of Chinese Medicine, Beijing 100029, China; ^5^Beijing Key Laboratory of TCM Syndrome and Formula, Beijing University of Chinese Medicine, Beijing 100029, China

## Abstract

Qingre Jiedu (QJ) recipe exerted significant cardioprotective efficacy against heart failure (HF), which is a growing health concern that continues to endanger patients' lives. To investigate the protective properties and mechanism of the QJ recipe, we established hydrogen peroxide (H_2_O_2_)-induced H9C2 cells and HF rats. The predicted targets and significant pathways of QJ against HF were collected and screened based on network pharmacology from key ingredients and validated by *in vivo* and *in vitro* experiments. The decoction of QJ (0.823 g/kg/day) was intragastrically administered for four weeks. QJ (400 *μ*g/mL) was cultured with H_2_O_2_ stimulated in the H9C2 cells. A total of 31 effective active compounds were screened in QJ and covered 277 targets, of which 85 were shared with HF-related targets. *In vivo*, the QJ recipe remarkably protected heart function and reduced serum IL-1, IL-6, PIIINP, and CIV levels. Furthermore, QJ downregulated the key proteins mediating inflammatory responses (p-IKK*α*/*β*, p-NF*κ*B, and IL-6) and cardiac fibrosis (STAT3 and MMP-9). *In vitro*, QJ protected the cardiomyocytes against H_2_O_2_-stimulated reactive oxygen species (ROS) production and upregulated PI3K and AKT expressions. Further experiments demonstrate that PI3K inhibitor LY294002 remarkably compromised the effects of QJ. In conclusion, our findings indicate that QJ could exert a cardioprotective effect and inhibit fibrosis and inflammation in HF rats via the PI3K-AKT signaling pathway.

## 1. Introduction

Despite recent advances in treating cardiovascular disorders, heart failure (HF) remains the leading cause of death worldwide, imposing a heavy social and economic burden [1]. Fibrosis and inflammation play a key part in response to HF pathological process [2]. Following fibrosis, a persistent proinflammatory reaction contributes to adverse post-MI left ventricular (LV) remodeling [3]. Herein, there is an urgent need to decipher the mechanism of fibrosis and inflammation in HF and identify new therapeutic targets for HF treatment.

During myocardial infarction, the necrotic cardiomyocytes release various danger-associated molecular patterns (DAMPs) and complement the components, which are neutralized by releasing cytokines like IL-6 [4]. IL-6 activates the PI3K-AKT pathway via its receptor (IL-6R*α*) [5]. PI3K belongs to a conserved family of lipid kinases and is the primary regulator of AKT activation [6]. PI3K could phosphorylate IKK into active form and activate downstream target NF-*κ*B to regulate inflammatory responses [7]. NF-*κ*B is a key transcriptional factor that regulates the expressions of inflammation-related genes, such as TNF-*α* and IL-6 [8]. Cytokines are released, which exacerbate inflammation response and other remodeling markers in myocardial fibrosis (MFs), such as MMP-9 [9]. Matrix metalloproteinase 9 (MMP-9) can degrade the extracellular matrix (ECM) components and play a critical role in myocardial fibrosis [10]. Additionally, the pieces of evidence have revealed that PI3K activation could decrease the level of signal transducer and activator of transcription 3 (STAT3) phosphorylation to reduce fibrosis [11]. STAT3 is involved in ECM degradation and has been demonstrated to be a crucial player in regulating myocardial fibrosis [12]. Besides, because of unbalanced deposition and degradation of extracellular matrix, collagen fibers replace the necrotic myocardial tissue, resulting in scar tissue accumulation, pathological remodeling of the myocardium, and decreased cardiac function [13].

Qishen Granule (QSG), a traditional Chinese medicine prescription, is a formula with significant efficacy in HF treatment [14, 15]. Additionally, according to Traditional Chinese Medicine (TCM) theory, the formula's ingredients should ideally achieve efficacy-oriented compatibility and mutual balance. Qingre Jiedu (QJ), from a recipe of QSG, is composed of two herbs (*Lonicera japonica* Thunb. [*Caprifoliaceae*] and *Scrophularia ningpoensis* Hemsl. [*Scrophulariaceae*]). Impressively, our earlier investigation demonstrated that QJ exhibited anti-inflammatory and antifibrosis effects in the postmyocardial infarction (MI) heart failure model [16]. However, accurate mechanisms by which QJ mitigates MI-induced inflammation and fibrosis remain unknown. This study probed and demonstrated drug-target relationships based on network pharmacology analysis and provided a reliable result of QJ recipe effects on HF using this method. In addition, we explored how QJ could ameliorate anti-inflammatory and antifibrosis effects and the potential mechanism.

## 2. Materials and Methods

### 2.1. Data Preparation

The components (oral bioavailability ≥30%, drug-likeness ≥ 0.18) of *Scrophularia ningpoensis* Hemsl. [*Scrophulariaceae*] (Xuanshen) and *Lonicera japonica* Thunb. [*Caprifoliaceae*] (Jinyinhua) identified in the QJ recipe were collected from TCMSP (http://ibts.hkbu.edu.hk/LSP/tcmsp.php), and the potential targets of QJ ingredients were predicted with an analogous method applied in the BATMANTCM (http://bionet.ncpsb.org/batman-tcm/) platform. For drug screening and evaluation, comprehensive information acquired from distinct databases on all herbal ingredients was used. Known targets related to HF were screened using “heart failure” as the keyword from the DisgenetGene database (http://www.disgenet.org). In addition, compound names were standardized according to PubChem CIDs (https://pubchem.ncbi.nlm.nih.gov/). Additionally, the obtained targets were committed to the website of UniProt (http://www.uniprot.org) to verify their gene names.

### 2.2. Network Construction and Analysis

Two QJ and HF gene lists were submitted to STRING (https://string-db.org/), and the species were only limited to “*Homo sapiens*” with a confidence score ≥0.7. The two PPI interactive networks were built and visualized using Cytoscape software (version 3.7.2, http://www.cytoscape.org/), and protein-protein interactions with a score ≥0.7 were gathered, followed by the generation of new PPI networks. After merging these two networks as a candidate network according to the intersection of PPI data, the QJ-HF PPI network was built. The topological features of these PPI networks were analyzed mainly based on degree, indicating the importance of nodes' biological function. To elucidate the functions of targets in signaling transduction, the KEGG signaling pathway analysis was used to interpret molecular function (MF), cellular component (CC), and biological process (BP) of key target genes of the QJ recipe in HF treatment.

### 2.3. Preparation of QJ and Quality Control

The QJ recipe samples were prepared using *Scrophularia ningpoensis* Hemsl. (*Scrophulariaceae*) (Xuanshen) and *Lonicera japonica* Thunb. (*Caprifoliaceae*) (Jinyinhua) by a weight ratio of 1 : 1. Xuanshen and Jinyinhua were obtained from Beijing TongRen-Tang Chinese Medicine Co. Ltd. (Beijing, China) and were identified by qualified experts. These two herbs were mixed and extracted three times as previously described [17].

### 2.4. Animals Experiments, Grouping, and Model Establishment

After one week of acclimation, Sprague-Dawley (SD) male rats (220 ± 10 g) obtained from Beijing Vital River Laboratory Animal Technology Co. Ltd. were randomly divided into three groups (*n* = 8): sham group, model group, and QJ-treatment group. Rats in the control group were subjected to sham surgery, whereas other rats underwent direct left anterior descending (LAD) artery ligation as described in our previous study [18]. Rats in the QJ group were administered with QJ at the dosage of 0.823 g/kg per day, whereas rats in the sham and model groups received the same volume of distilled water. All drugs and distilled water were gavaged with the amount of *l* mL/100 g for 28 days. Our study followed the Guide for the Care and Use of Laboratory Animals established by the US National Institutes of Health (NIH publication No. 85–23, revised 1996), and it was approved by the Ethics Committee of Experimental Animals of Beijing University of Chinese Medicine (BUCM-3-2016040201-2003).

### 2.5. Echocardiographic Assessment

Echocardiographic measurements were used to assess heart functions (Vevo 2100, Visual Sonics, Canada). The echocardiography was performed under general anesthesia with 1% pentobarbital sodium. M-mode tracks were recorded using the anterior and posterior walls of the left ventricular (LV) at the papillary muscle level. Heart functions were assessed using related parameters, including ejection fraction (EF), fractional shortening (FS), left ventricular internal dimension-diastole (LVID; d), and left ventricular internal dimension-systole (LVID; s). Three consecutive cardiac cycles were recorded during measurements.

### 2.6. Histological Examination

The rat cardiac tissues were cut horizontally using the mid-region to create cross-sections of the left and right ventricles, and the apex part was fixed with 4% paraformaldehyde. After embedding the tissues in paraffin, they were sectioned into 5 *μ*m slides. After deparaffinization with xylene and rehydration via different grades of ethanol, the sections were stained with hematoxylin-eosin (HE), Masson, and Sirius-red to assess overall pathological changes. The images were visualized under an optical Leica Biosystems Richmond microscope at 400 magnification.

### 2.7. Measurement of IL-1, IL-6, PIIINP, and CIV Levels

The serum was collected from the fresh blood and centrifuged at 3000 g for 10 min at 4°C. The concentrations of serum interleukin-1 (IL-1), interleukin-6 (IL-6), procollagen III propeptide (PIIINP), and collagen IV (CIV) were detected using enzyme-linked immunosorbent assay according to the manufacturer's protocol.

### 2.8. Cell Culture and Cell Viability

The H9C2 cardiomyocytes were purchased from the National Experimental Cell Resource Sharing Service Platform (Beijing, China) and incubated in high-glucose Dulbecco's modified Eagle's medium (DMEM, Hyclone, United States) supplemented with 10% fetal bovine serum (FBS, Corning, United States) and a mixture of 1% penicillin/streptomycin in a humidified atmosphere with 5% CO_2_ at 37°C. To establish the *in vitro* inflammatory injury model, the H9C2 cells were stimulated with H_2_O_2_. The H9C2 cells were grown with QJ for 24 h until they reached 80% to 90% confluence, and the cells were stimulated with H_2_O_2_ for 4 h with or without QJ to evaluate the cytotoxicity and effects of QJ in the H9C2 cells. The cells were cultured in 96-well plates at a density of 6 × 10^3^ cells/well and treated with QJ at different concentrations (200, 400, 600, 800, and 1000 *μ*g/mL). For subsequent experiments, the cells were randomly divided into four groups: control group, H_2_O_2_-stimulated model group, QJ group, and QJ + LY294002 group.

### 2.9. Detection of Reactive Oxygen Species Detection

ROS was measured by a commercial assay using fluorescent probe DCFH-DA. The H9C2 cells were stimulated with H_2_O_2_ with or without QJ and LY294002 and observed at excitation and emission wavelengths of 488 and 525 nm under a fluorescence microscope (Leica Microsystems GmbH).

### 2.10. Western Blot Analysis

The myocardial tissues and H9C2 cells were homogenized in RIPA lysis buffer and quantified using bicinchoninic acid (BCA) method. Protein samples (50 *μ*g per sample) were separated using 10% sodium dodecyl sulfate polyacrylamide gel electrophoresis (SDS-PAGE) and then electrotransferred to a PVDF membrane. After blocking in TBST containing 5% skimmed milk for 1.5 h at room temperature, the membranes were incubated at 4°C overnight with the following primary antibodies: anti-p-NF*κ*B (ab97726; Abcam, United States), NF-*κ*B (CST8242, Cell Signaling Technology, Germany), anti-IL-6 antibody (ab208113; Abcam, United States), anti-p-STAT3 (ab76315; Abcam, United States), STAT3 (ab68153; Abcam, United States), MMP-9 (ab38898; Abcam, United States), PI3K (ab182651; Abcam, United States), AKT (ab182729; Abcam, United States), p-AKT (CST4060, Cell Signaling Technology, Germany), p-IKK*α*/*β* (CST2697 T, Cell Signaling Technology, Germany), IKK*α* (CST2682, Cell Signaling Technology, Germany), IKK*β* (CST8943, Cell Signaling Technology, Germany), and anti-GAPDH (ab8245, Abcam, 1 : 5,000). Following that, the membranes were washed and incubated with specific horseradish peroxidase (HRP)-conjugated secondary antibodies (goat anti-rabbit IgG 1 : 12,000 and goat anti-mouse IgG 1 : 5,000) for 1 h. The blots were visualized with Omni ECL reagent (EpiZyme, China) for 1 min at room temperature. They were captured and analyzed using UVP BioImaging Systems (Bio-Rad, Hercules, CA, United States). Furthermore, protein expressions were normalized based on GAPDH level, and grayscale analysis was performed using Image-LAB software.

### 2.11. Statistical Analysis

SPSS 22.0 or GraphPad Prism 7 were used for statistical analysis. All data were presented as mean ± standard deviation (SD). Dunnett's test and one-way analysis of variance (ANOVA) were employed to compare differences among multiple groups. *P* values less than 0.05 were considered statistically significant.

## 3. Results

### 3.1. Efficacy Evaluation after QJ Recipe Treatment in the HF Model

**Figure 1:**
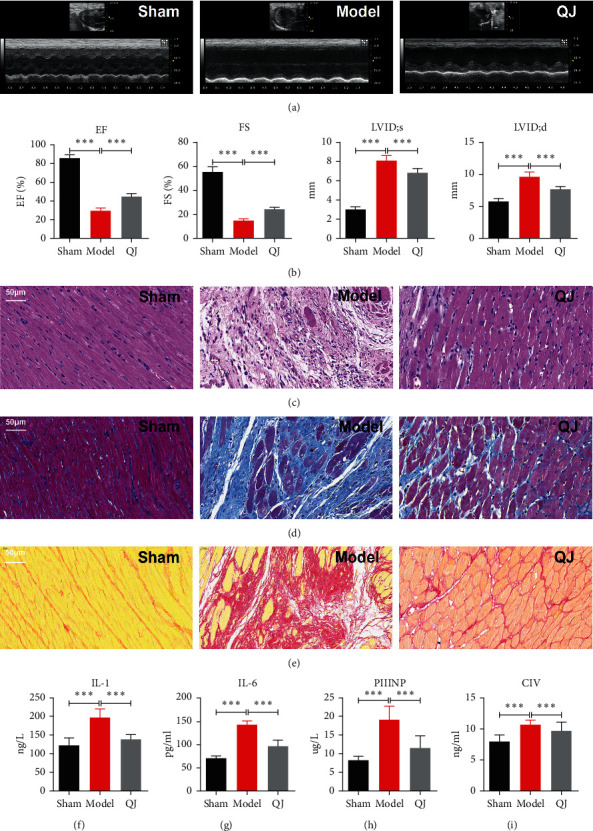
Efficacy evaluation after QJ recipe treatment. (a) Echocardiography was conducted to detect cardiac function in sham, model, and QJ groups. (b) Echocardiography data showed that QJ treatment could increase EF and FS values and decrease LVID; s and LVID; d values. (c) HE staining showed that QJ treatment alleviated inflammatory responses, scale bar = 50 *μ*m. (d, e) Masson staining and Sirius-red staining showed that QJ treatment attenuated collagen deposition and fibrotic remodeling, scale bar = 50 *μ*m. (f–i) Levels of IL-1, IL-6, peptide of type III procollagen (PIIINP), and collagen IV (CIV) in serum. QJ treatment could alleviate inflammatory responses and reduce myocardial fibrosis. Data were presented as the mean ± standard error of three independent experiments (*N* = 8 per group; ^*∗∗*^*P* < 0.01, ^*∗∗*^*P* < 0.001).

After 28 days of QJ treatment, cardiac function was examined using echocardiography. Echocardiography data revealed that rats in the model group had significantly lower values of EF and FS, while their diameters on LVID; s and LVID; d were longer than those in the sham group, revealing that the HF model was successfully induced. Besides, both EF and FS were upregulated compared to the model group, implying that QJ treatment improved left ventricular function. Additionally, LVID; s and LVID; d in the QJ group were reduced than those in the model group (Figures 1(a) and 1(b)).

Following that, myocardial inflammation was evaluated. HE staining demonstrated that myocardium was normal, and cardiomyocytes were neatly arranged in the sham group. However, the HF model group revealed massive necrosis and cardiomyocyte derangement. Besides, a large number of normal structural loss and infiltrating inflammatory cells were observed. Hearts were rescued from inflammatory cell infiltration and maintained their original morphology following QJ treatment (Figure 1(c)). Furthermore, the serum levels of IL-1 and IL-6 were higher in the rats of the model group than those in the sham group, however, the QJ treatment reversed the changes (Figures 1(f) and 1(g)). These findings indicated the effects of QJ on cellular inflammation injury.

Next, we evaluated fibrosis. Masson and Sirius-red staining demonstrated a marked increase in collagen content in the model group. QJ treatment could greatly reduce the contents of collagen deposition and fibrotic remodeling compared with the model group (Figures 1(d) and 1(e)). Likewise, the serum peptides of type III procollagen and collagen IV levels in the model group were higher than those in the sham group, however, QJ treatment evidently alleviated them (Figures 1(h) and 1(i)). These findings established the presence of cardiac fibrosis and demonstrated that QJ could alleviate myocardial fibrosis and protect hearts from fibrotic remodeling.

### 3.2. Potential Targets of QJ Recipe in HF Treatment and KEGG Pathway Enrichment Analysis of Common Targets

**Figure 2:**
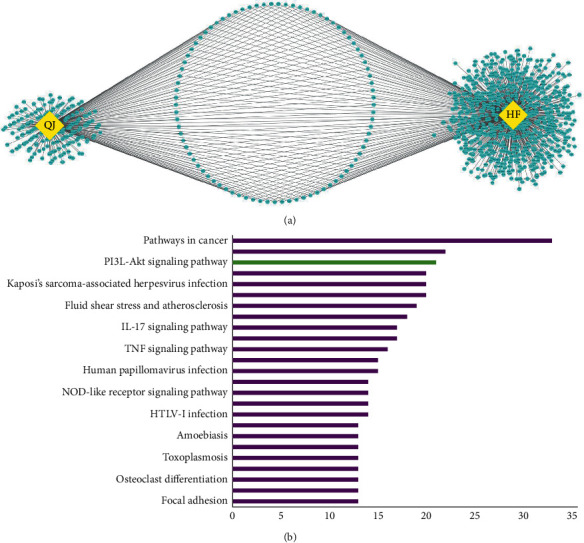
Potential therapeutic targets of QJ recipe in the treatment of HF and KEGG pathway analysis of common targets. (a) Network of QJ and HF-related targets. The nodes on the left indicate the associated targets of QJ and those on the right represent potential HF-related targets from the DisgenetGene database, whereas the nodes in the middle represent the core targets of QJ in the treatment of HF. The edges represent correlations between the targets. (b) The top 25 pathways measured by gene count are selected to reveal the key biological actions of major hubs. The abscissa represents the number of genes associated and the ordinate indicates main pathways. The raw data are listed in Supplementary Table 2.

The bioactive components and potential targets of Xuanshen and Jinyinhua, which constitute the QJ recipe, were identified and listed in Supplementary Table 1. In QJ, 31 effective active compounds were identified and covered 277 targets, 85 of which were shared with HF-related targets (Figure 2(a)). To further investigate the effects of common targets, we performed the KEGG pathway enrichment analysis, as depicted in Figure 2(b). Biological pathway analyses indicated that QJ was enriched in several signal pathways. Among them, the PI3K-Akt signaling pathway was significantly enriched. Therefore, the PI3K-Akt signaling pathway may be a potential pathway of QJ for anti-inflammatory and antifibrosis mechanisms.

### 3.3. Protein-Protein Interaction (PPI) Networks Construction

**Figure 3:**
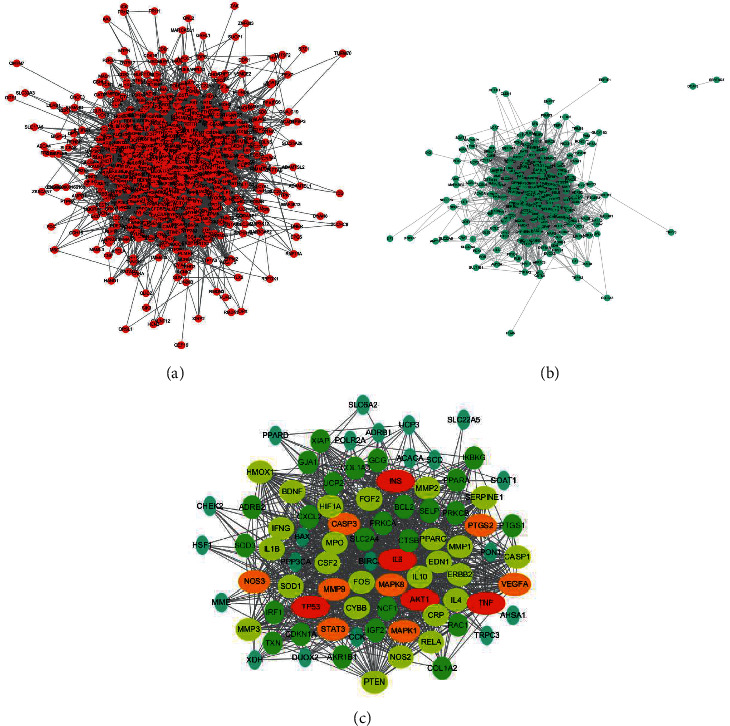
PPI networks construction. (a) PPI network of nodes regulated by the QJ recipe. (b) PPI network of nodes associated with the therapeutic mechanism of HF. (c) Core targets regulated by the QJ recipe in the treatment of HF with 85 nodes and 1166 edges: the nodes suggest genes or targets, the edges indicate the interactions between the nodes, and the size of the nodes represents the value of the degree. The raw data are listed in Supplementary Table 3.

All QJ and HF core gene names were converted to STRING, respectively, and QJ and HF-related target networks were constructed with PPI databases. The results revealed that the HF PPI network consisted of 762 proteins and 14,639 interactions, whereas 268 proteins and 4650 interactions were included in the QJ PPI network (Figures 3(a) and 3(b)). Furthermore, the interactive QJ-HF PPI network was finally obtained after merging the above two PPI networks. The results indicated that the merged network consisted of 85 core targets, corresponding to the crucial targets regulated by the QJ recipe in HF treatment (Figure 3(c)).

### 3.4. The Pharmacological Mechanisms of QJ Recipe against HF

**Figure 4:**
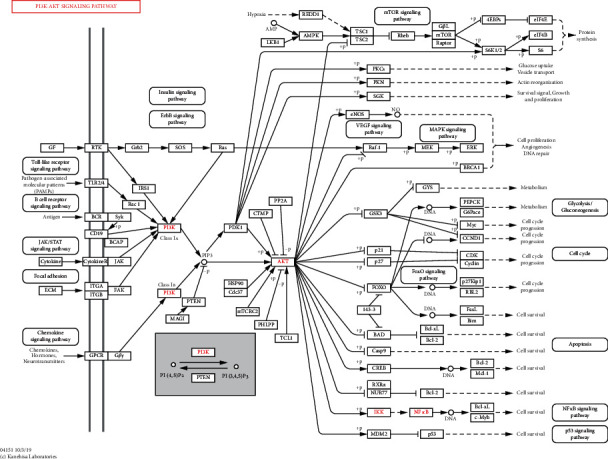
The pharmacological mechanisms of how the QJ recipe treats HF. The PI3K-Akt signaling pathway plays a crucial role in the treatment of HF. The targets in red could be considered important in the mechanism diagram of PI3K-Akt signaling pathway.

To further investigate the potential pharmacological mechanism, we screened the top 20 hub genes in common targets based on the betweenness centrality and the degree of PPI network analysis (Supplementary Table 4). The results indicated that hub genes, such as AKT, IL-6, STAT3, and MMP-9, are critical in HF treatment. As a result, these key targets related to the PI3K-Akt signaling pathway may exert significant influence in HF treatment (Figure 4).

### 3.5. Effects of QJ on Myocardial Inflammatory and Fibrosis via PI3K/AKT-IKKs/STAT3 Pathway

**Figure 5:**
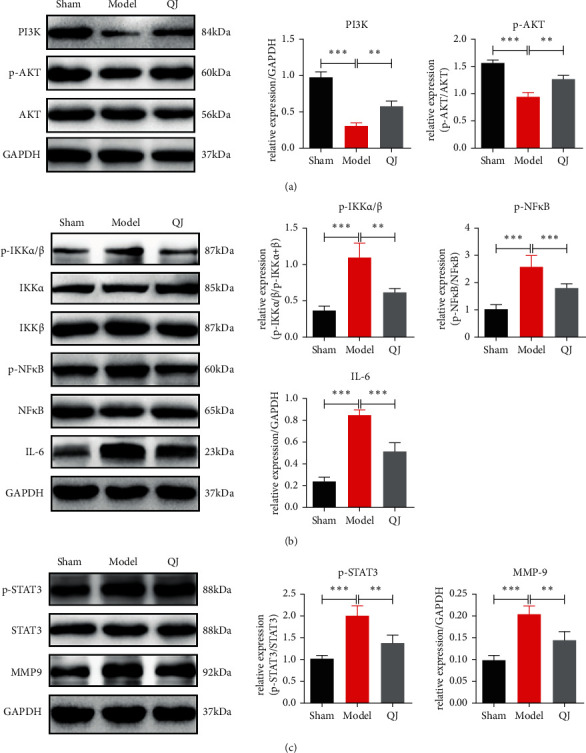
Effects of QJ on myocardial inflammatory and fibrosis via PI3K/AKT-IKKs/STAT3 pathway. (a) Western blots assessed the expressions of PI3K, p-AKT, and AKT in HF rats, and QJ could upregulate the expressions of PI3K and p-AKT. (b) Western blot bands of p-IKK*α*/*β*, p-NF*κ*B, and IL-6 and their quantitative results in HF rats, and QJ could downregulate the expressions of p-IKK*α*/*β*, p-NF*κ*B, and IL-6. (c) Western blot bands of p-STAT3 and MMP-9 and their quantitative results in HF rats, and QJ could downregulate the expressions of p-STAT3 and MMP-9. Data were presented as the mean ± standard error of three independent experiments (*N* = 3 per group; ^*∗∗*^*P* < 0.01, ^*∗∗*^*P* < 0.001).

PI3Ks are kinases that respond to different types of membrane receptors, which have been implicated in developing various cardiovascular diseases, such as heart failure, thrombosis, atherosclerosis, and hypertension [19]. Network pharmacology analysis revealed that the PI3K-AKT signaling pathway is involved in the QJ anti-inflammatory and antifibrosis effect in HF. Western blotting results revealed that the protein level of PI3K was significantly downregulated in the model group, however, QJ treatment reversed the changes. p-AKT expression in the model group was decreased. After treatment with QJ, p-AKT expression was increased remarkably (Figure 5(a)). The IKKs-NF*κ*B pathway is considered the initiation of the inflammatory cascade, and there is increasing evidence that IL-6-mediated inflammation response exacerbates adverse remodeling post-MI [20]. To further confirm the role of QJ in inflammation, the critical proteins were detected by western blotting. The results indicated that IKK*α*/*β* and NF-*κ*B expressions remained relatively unaltered among different groups, whereas those of phosphorylated IKK*α*/*β* (p-IKK*α*/*β*) and NF-*κ*B (p–NF–*κ*B) (active forms of IKK*α*/*β* and NF-*κ*B) increased significantly in the model group compared with the sham group. Additionally, p-IKK*α*/*β* and p-NF*κ*B expressions were reduced significantly by QJ treatment (Figure 5(b)). IL-6 is a target gene for NF-*κ*B. Consistently, IL-6 levels in the model group were increased compared with those in the sham group, whereas QJ treatment partly reversed IL-6 expression (Figure 5(b)).

STAT3 and MMP-9 are implicated in ECM degradation [21,22]. By observing the effect of QJ on fibrosis in Figure 4, we detected the key proteins to degrade ECM. The results revealed that the expressions of phosphorylated STAT3 (p-STAT3) and MMP-9 were upregulated in the model group, and QJ impressively inhibited p-STAT3 and MMP-9 expressions compared with those in the model group (Figure 5(c)).

### 3.6. Effects of QJ on PI3K-AKT Signaling Pathway

**Figure 6:**
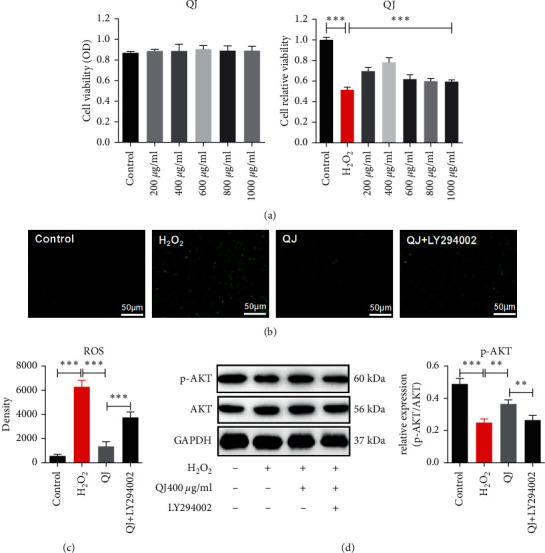
Effects of QJ on PI3K-AKT signaling pathway. (a) QJ (200, 400, 800, and 1000 mg/mL) has no toxicity on the H9C2 cells and increases cell viability remarkably in the H_2_O_2_-stimulated H9C2 model. (b) (c) The production of ROS on H_2_O_2_-stimulated H9C2 cells. (d) Western blotting detected the expressions of p-AKT in H9C2 cells. Data were presented as the mean ± standard error of three independent experiments (*N* = 3 per group for WB and *N* = 6 per group for the other experiments; ^*∗∗*^*P* < 0.001).

To further demonstrate the mechanism of QJ, we established an H_2_O_2_-induced H9C2 cell injury model. As illustrated in Figure 6(a), treatment with 200–1000 mg/mL QJ proved to be effective. The dose of 400 mg/mL QJ proved to be the most effective dose, which was applied in subsequent experiments. In addition, ROS staining revealed that the protective effect of QJ was remarkably counteracted by PI3K inhibitor LY294002 in the H_2_O_2_-stimulated H9C2 cell injury model (Figure 6(b) and 6(c)).

PI3K and AKT are upstream proteins involved in regulating inflammation and fibrosis using IKKs/STAT3 signaling pathway [23]. To validate whether QJ anti-inflammatory and antifibrosis effects were mediated by PI3K activation, we detected p-AKT expression in the H_2_O_2_-stimulated H9C2 cell injury model and used the inhibitor of PI3K, LY294002, *in vitro.* QJ treatment activated p-AKT expression. More importantly, the cotreatment of *β*-elemene with PI3K inhibitor LY294002 failed to reverse its activation (Figure 6(d)), indicating that QJ protected H_2_O_2_-stimulated the H9C2 cells against the injury partly mediated by the PI3K-AKT pathway.

## 4. Discussion

In this study, network pharmacology and experimental validation *in vivo* and *in vitro* were conducted to investigate the cardioprotective effects of QJ against HF and its underlying mechanism. Our main findings are as follows: (1) QJ revealed cardioprotective effects in HF rats' model. (2) QJ decreased inflammatory response and inflammation-induced fibrosis. (3) The anti-inflammation and antifibrotic effects of QJ may be mediated by the PI3K-AKT signaling pathway.

Network pharmacology is a promising method for elucidating the mechanisms underlying Chinese herbal formulas [24]. In this study, 762 potential targets related to HF were identified based on the network pharmacology approach. We found that the effects of QJ in treating HF involved several major signaling pathways. More importantly, the network pharmacological analysis revealed that QJ could improve heart functions using the PI3K-AKT signaling pathway. The accumulated pieces of evidence indicate that PI3K-AKT intersects with many different inflammatory signaling pathways, including the NF-*κ*B pathway [25]. These processes were involved in myocardial fibrosis [26].

Extensive experimental evidence indicates that MI is closely related to inflammatory reaction activation and subsequent inflammation-induced fibrosis [27]. At the onset of MI, cardiac resident cells and circulating inflammatory cells produce cytokines, such as IL-6 [28]. Active inflammatory factors cause inflammation and fibrosis in a vast area [29]. Excessive fibroblast activation may contribute to fibrotic area enlargement, promoting diastolic dysfunction and increasing myocardial stiffness [30].

Research has revealed that *Lonicera japonica* and *Scrophularia ningpoensis*, as representative drugs for the Qingre Jiedu recipe, show therapeutic effects on a variety of inflammation and progressive fibrosis-related diseases, including atherosclerosis, melanoma, myocardial ischemia-reperfusion injury, pelvic inflammation, rheumatoid arthritis, nonalcoholic steatohepatitis, liver fibrosis, lung fibrosis, etc. [31–34]. Moreover, Si-Miao-Yong-An decoction (SMYAD), comprising QJ important recipes, has also been demonstrated to have an antifibrosis effect [35]. More important, the QSG formula may protect against heart failure via several mechanisms, such as energy metabolism, apoptosis, inflammation, fibrosis, oxidative stress, etc. [36–39]. In general, *Lonicera japonica* and *Scrophularia ningpoensis* may have a protective effect on myocardial fibrosis mediated by inflammation after myocardial infarction. However, the biological function and therapeutic mechanisms of decomposed recipes of QSG on HF remained unclear, which is crucial for understanding compatibility and efficacy regularity for further development of QSG formulas. In this work, we demonstrated that QJ exhibits an effective anti-inflammatory impact and inhibits inflammation-induced fibrosis. Furthermore, in our study, we confirmed that EF and FS values were decreased in this model. After treatment with QJ, the EF and FS values were upregulated, implying that QJ had cardioprotective effects in this model. HE and Masson staining revealed that inflammation and fibrosis appeared in this model. In addition, the level of IL-6 in the model group elevated dramatically compared with the sham group. Furthermore, QJ decreased collagen deposition and IL-6 levels *in vivo*, implying that QJ had a potential effect on anti-inflammation and a decreased degree of myocardial fibrosis.

To elucidate the underlying mechanism of its anti-inflammation and antifibrosis, we used network pharmacology to investigate the targets of QJ in treating HF. In recent years, network pharmacology has gained widespread attention because of its ability to integrate numerous fields, such as bioinformatics, electronic technology, molecular biology, and pharmacology, to establish network relationships among the active compounds of diseases, Chinese formulas, pathways, and relevant targets [40]. Biological pathway analyses indicated that QJ enriched the targets of several signal pathways. Among them, the PI3K-AKT signal pathway was significantly enriched, implying its protective effects through PI3K/AKT signaling activation. PI3K/AKT pathway has been implicated in the fibrosis process by affecting fibroblast differentiation and modulation of massive downstream factors, such as NF-*κ*B [41, 42]. Our study revealed that QJ could increase PI3K and AKT expressions while decreasing p–NF–*κ*B, p-STAT3, and MMP9 levels.

Furthermore, to investigate the cardiac protective effect against HF, we conducted comprehensive research on inflammatory responses and cardiac fibrosis. The results indicated that QJ could downregulate the protein levels of p-IKK*α*/*β*, p-NF*κ*B, and IL-6, demonstrating that QJ has a regulative efficacy on the anti-inflammation pathway. Additionally, QJ demonstrated a regulative effect on myocardial fibrosis. QJ decreased p-STAT3 and MMP-9 expressions while also downregulating the levels of myocardial type I and III collagen. These results indicated that QJ could alleviate inflammatory responses and attenuate cardiac fibrosis to exert a myocardial protective effect against HF. The upstream pathways that mediate inflammation and fibrosis were further validated. QJ could upregulate PI3K expression and activate p-AKT expression significantly.

ROS is a type of DAMPs released by the necrotic cardiomyocytes that could activate inflammation [43]. In addition, ROS has been demonstrated to regulate collagen metabolism in the cardiac fibroblasts by directly affecting the synthesis and activity of the degradative enzymes [44]. Therefore, we established an H_2_O_2_-induced H9C2 cell injury model to investigate relevant mechanisms of QJ *in vitro.*

In our study, QJ was found to be effective against H_2_O_2_-induced loss of cell viability and reduced ROS production. In addition, QJ increased p-AKT in this model. After cotreatment with LY294002, the inhibitor of PI3K could reduce p-AKT expressions, implying that QJ could activate PI3K to regulate inflammation-induced fibrosis. To further investigate the effect of QJ on the PI3K-AKT pathway, the inhibitor of PI3K was combined with QJ. Western blotting revealed that increasing p-AKT expression was abrogated by LY294002 in the QJ group, illustrating that QJ exerted cardioprotection by the PI3K-AKT pathway.

As a limitation of our research, the effective compounds of QJ that participated in mediating anti-inflammation and fibrosis pathways mediated by PI3K-AKT remain unclear. Furthermore, we will probe the combination of effective compounds and PI3K targets in QJ to interpret the regulatory mechanism of the QJ recipe more comprehensively and systematically.

## 5. Conclusion

**Figure 7:**
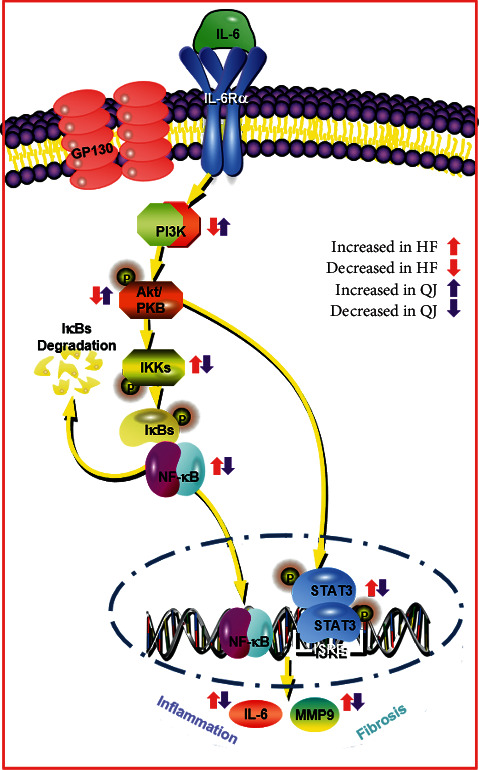
Underlying mechanism of QJ on anti-inflammatory and antifibrosis in heart failure.

QJ exerts *in vivo* and *in vitro* anti-inflammatory and fibrotic effects, which may be regulated by the PI3K-AKT signaling pathway (Figure 7). These findings can further improve our comprehension of the therapeutic mechanisms underlying the QJ decomposed recipes against HF.

## Data Availability

The data used to support the findings of this study are available from the corresponding author upon request.
