# Supplementary material for: Mechanistic Insights of Qingre Jiedu Recipe Based on Network Pharmacology Approach against Heart Failure
Source: Evid Based Complement Alternat Med. 2022 Jan 31;2022:9024394. doi: 10.1155/2022/9024394 (PMC8820871; doi:10.1155/2022/9024394)
Supplement: Supplementary Materials — Supplementary material related to this article can be found in Supplementary Tables 1, 2, 3, and 4. [file 9024394.f1.zip › 9024394.f1/Supplementary Table 1.docx]

**Supplementary Table 1 . Components and targets of QJ recipe.**

| Herb | Component | Target |
| --- | --- | --- |
| Scrophularia ningpoensis Hemsl. [Scrophulariaceae] | Harpagoside, paeoniflorin,  14-deoxy-12 (R) sulfoandrographolide, scropolioside A, beta sitosterol, sitosterol, scropolioside D, sugiol | ACHE，ADRA1A，ADRA2A，ADRB1，ADRB2，AKR1B1，ANTXR2，APAF1，ATF2，BCL2，BDNF，BIRC5，BTK，CA2，CASP1，CASP3，CASP8，CCND2，CDK6，CETP，CHRM1，CRAT，CREB1，CRP，CSF2，CTSB，CTSD，DNPEP，DUOX2，EDN1，ENPEP，ERBB2，F3，FASLG，FGF2，GABBR1，GFAP，GOT1，GRIA2，GSTM1，GSTM2，HMGCR，ICAM1，IGF2，INPPL1，INS，IRF1，JUN，LITAF，LPL，MAOA，MAOB，MAP2，MAPK8，MAPK8IP1，MCL1，MGAM，MMP10，MMP3，MPO，NOS3，NTRK2，PAM，PDX1，PLAU，PLG，PLK1，PON1，PPARA，PPARD，PRKCA，PRKCG，PTGER3，PTGS1，PTGS2，PTPN1，RAC1，RELA，SELE，SELP，SERPINE1，SLC2A2，SLC6A2，SLC6A3，SOAT1，SOD1，SRC，STAT1，STAT3，TNF，TYRP1，UCP2，CCK，CCNB1，CDC25C，BAX，CASP9，GAP43，GCG，FABP1，SFN，PTPN6，SCD，SELL，POLR2A，RBP2，CITED1，PECAM1，PYY，UCP3，TEP1，MAPK8IP2，HERC5，ENPP7 |
| Lonicera japonica Thunb. [Caprifoliaceae] | Ethyl linolenate, phytofluene, Eriodyctiol (flavanone), (-)-(3R,8S,9R,9aS,10aS)-9 ethenyl-8-(beta-D-glucopyranosyloxy) 2,3,9,9a,10,10 ahexahydro-5-oxo-5H,8H-pyrano[4,3-d] oxazolo [3,2-a] pyridine-3-carboxylic acid_qt, secologanic dibutylacetal, beta-carotene, ZINC03978781, Chryseriol, kryptoxanthin,4,5'-Retro-.beta.,.beta.-Carotene-3,3'-dione, 4',5'-didehydro-5-hydroxy-7-methoxy-2-(3,4,5-trimethoxyphenyl)chromone,7-epi Vogeloside, CaerulosideC, Centauroside, Ioniceracetalides B, XYLOSTOSIDINE, dinethylsecologanoside, beta-sitosterol, kaempferol, Stigmasterol, luteolin, quercetin | ALOX5，AMY2A，APC，BCL2，BCL2L1，BIRC5，CA2，CAPN1，CASP1，CASP3，CASP7，CCND1，CCND2，CD40，CD40LG，CD86，CDK4，CDK6，CDKN1A，CDKN2A，CHEK2，CHRM1，COL1A1，COL1A2，CRYZ，CXCL10，CXCL8，CYP1A1，CYP1A2，CYP1B1，CYP3A4，DUOX2，EIF6，ENOX2，EP300，ERBB2，ERBB3，F3，FOS，FXYD2，G6PC，GCLC，GJA1，GLS2，GRIA2，GSTM1，GSTM2，GSTP1，HIF1A，HIRA，HK1，HMGCR，HMOX1，HSF1，ICAM1，IFNG，IGF1R，IGFBP3，IGHG1，IKBKG，IL10，IL1B，IL2，IL4，IL6，INPPL1，INS，INSR，IRF1，ITGB2，JUN，KAT5，LCAT，LCT，MAOA，MAOB，MAPK1，MAPK3，MAPK8，MAPK8IP1，MCL1，MDM2，MGAM，MME，MMP1，MMP10，MMP2，MMP3，MMP9，MS4A2，MT3，MUC1，MYC，NCF1，NFKBIA，NOS2，NOS3，NPEPPS，NQO1，NR1H4，NR1I3，ODC1，PCNA，PCYT1A，PLAU，PON1，POR，PPARA，PPARD，PPARG，PPP3CA，PRKCA，PRKCB，PRKCE，PRKCG，PRKCZ，PRODH，PRSS1，PTEN，PTGER3，PTGS1，PTGS2，PTPN1，RASA1，RASSF1，RB1，RELA，RUNX2，RXRA，SELE，SERPINE1，SI，SLC22A5，SLC37A4，SLC5A5，SLC6A2，SOD1，STAT1，SULT1E1，TBXA2R，THBD，TLR2，TNF，TOP1，TOP2A，TP53，TRPC3，TRPV3，TXN，TYR，VCAM1，VEGFA，XDH，XIAP，CCNB1，BAK1，BAX，CHI3L1，DIO1，ALPI，E2F2，AMY1C，CLDN4，CASP9，RUNX1T1，HAS2，HK2，IVL，FABP1，HSPA1B，CXCL2，IGHD，PTPN6，NKX3-1，CCL20，CXCL11，RBP2，PGA5，PSMD3，SREBF2，TRPM2，SLC2A4，SLPI，SOD3，AHSA1，PSME3，DCAF5，EEF1E1，ABI1，MAPK8IP2，SERTAD3，ALG5，HERC5，CUTA，NUF2，TRIM63，FBXO32，PLB1，ENPP7，GUSBP1，CASP12，PRXC1A，GUCY1B3，gyrB，CDC2，DEFB4，CYBB |
