# Supplementary material for: Mechanistic Insights of Qingre Jiedu Recipe Based on Network Pharmacology Approach against Heart Failure
Source: Evid Based Complement Alternat Med. 2022 Jan 31;2022:9024394. doi: 10.1155/2022/9024394 (PMC8820871; doi:10.1155/2022/9024394)
Supplement: Supplementary Materials — Supplementary material related to this article can be found in Supplementary Tables 1, 2, 3, and 4. [file 9024394.f1.zip › 9024394.f1/Supplementary Table 2.docx]

**Supplementary Table 2. Potential targets of heart failure.**

| **Heart Failure Related-Targets** |
| --- |
| SERPINA3, ABCA4, ACACA, ACLY, ACTC1, ADCY6, ADD1, ADM, ADORA2A, PARP1, ADRA2C, ADRB1, ADRB2, ADRB3, GRK2, GRK3, ACAN, AGER, AGT, AGTR1, AGTR2, APLNR, AKT1, ALAS2, ALB, ALDH2, AKR1B1, ALOX12, ALOX15, AMPD1, BIN1, ANG, ANGPT1,ANGPT2,ANK2, ANXA2, ANXA5, ANXA6, APCS, XIAP, BIRC5, APOA1, APOC1, APOE, APRT, AQP2, ABCC6, ARG1, RND3, ARRB1, ARRB2, ZFHX3, ATF3, ATM, ATP1A3, ATP2A1, ATP2A2, ATP2B1, ATP2B4, ATP5F1A, AVP, AVPR1A, AVPR2, AXL, BAD, BAX, BCL2, HCN2, BDNF, BMP4, BMPR1A, BMPR2, BNIP3, BNIP3L, BRCA1, VPS51, DDR1, CAD, CALCR, CALM1, CALM2, CALM3, CALR, CAMK2D, CAST, CASP1, CASP3, CASQ2, CAT, CAV3, CCK, CD36, SCARB2, CD44, CD68, CD69, CDH15, CDK9, CDKN1A, CDS1, CHGA, CHRM4, CHRNA4, CIDEA, CISH, CLCN3, CLCNKA, CLIC2, CMA1, CCR7, CNN1, CNP, CNR2, COL1A1, COL1A2, COL11A2, CP, CPT1A, CPT2, CRK, CRMP1, CRP, CS, MAPK14, CSF1, CSF2, CSF3, CST3, CTF1, CTGF, CTSB, CX3CR1, CYBB, CYP2B6, CYP2D6, CYP2E1, CYP2J2, CYP11B1, CYP11B2, DAG1, DBH, DCN, ACE, DECR1, DES, CFD, DIO3, DMD, DNAH8, DNASE1, DNM2, DNMT1, DPP4, DPYD, DPYS, DSP, DTNA, DUSP1, ECE1, S1PR1, EDN1, EDNRA, EDNRB, EGFR, ELN, CTTN, ENDOG, ENG, EPHA3, EPHX2, EPO, ERBB2, ERBB4, EYA4, ERN1, ESRRA, F2RL1, FABP4, PTK2B, FASN, FBN1, FDPS, FGF2, FGF4, FKBP1B, FOXC1, FOXM1, FLNA, FLNC, FOS, FXN, MTOR, FRZB, FTH1, G6PD, XRCC6, GAB1, GABPA, GATA4, GATM, GC, GCG, GCKR, OPN1MW, MSTN, GH1, GHSR, GJA1, GLA, GLB1, GLP1R, GNAQ, GNB3, GOLGB1, GPR17, GRK5, GPT, GPX4, NR3C1, CXCL2, GSK3B, GSN, GSR, HADHA, HADHB, HBA1, HBA2, HCRTR2, HTT, HFE, HGF, NRG1, HIF1A, HMGB1, HMOX1, NR4A1, HNRNPD, TLX2, HP, HRC, HSF1, HSPA4, HSPB1, HTR2A, HTR2B, HTR4, TNC, ID2, IDS, IFNG, IGF1, IGF2, IGFBP4, IL1A, IL1B, IL4, IL6, IL6R, IL6ST, IL10, IL13RA1, IL16, IL17A, IL18, ILF3, ILK, INS, IRF1, ISL1, ITGB1, ITPK1, ITPR1, ITPR2, JARID2, JUP, KCNE1, KCNH2, KCNJ5, KCNK2, KNG1, LCN2, LEP, LGALS3, LIF, LIFR, LIPC, FADS1, LMNA, LOX, LPA, LUM, LY75, SMAD1, MAS1, MDH2, MECP2, MEF2A, MEF2C, MITF, NR3C2, MME, MMP1, MMP2, MMP3, MMP8, MMP9, MMP12, MMP13, MMP14, MPO, TRNC, COX1, COX2, COX3, CYTB, ND1, ND5, ND6, TRNE, TRNF, TRNK, TRNL1, TRNQ, TRNS1, TRNS2, TRNT, TRNV, TRNW, MUC2, TRIM37, MYBPC3, MYH6, MYH7, MYLK, MYOD1, NCAM1, NDUFS6, NFATC2, NFATC4, NFE2L1, NFE2L2, NFKB1, NGF, NGFR, NM, NME3, NOS1, NOS2, NOS3, CNOT3, NPY, NOTCH3, NPPA, NPPB, NPPC, NPR1, NPR2, NRF1, OGN, OLR1, OPA1, TNFRSF11B, OPRM1, P2RX1, P2RX4, P2RX7, P2RY2, P4HB, PEBP1, SERPINE1, PCK1, CDK16, PDC, PDE3A, PDE9A, PDK1, PDK4, ENPP1, PDPK1, PEX7, PGF, PGK1, ABCB1, PHYH, PIK3CA, PIK3CB, PIM1, PIK3CD, PIK3CG, PIN1, PITX2, PKD1, PKD2, PKP2, PLAT, PLCD1, PLCG1, PLD2, PLN, PLOD1, POLR2A, POMC, PON1, PPA1, PPARA, PPARD, PPARG, MED1, PPP1R1A, PPP2R1A, PTPA, PPP3CA, NPY4R, PRH1, PRH2, PRKACG, PRKAR1A, PRKAR2B, PRKCA, PRKCB, PRKD1, MAPK1, MAPK8, MAPK9, MAP2K3, MAP2K7, PRL, PSEN1, PSEN2, PSMB8, PTEN, PTH, PTGS1, PTGS2, PTHLH, PTN, PTX3, PURB, RAB1A, RAC1, MOK, RBP4, RELA, REN, RENBP, RET, RGS4, GRK1, ROCK1, RPL32, RPS19, RYR1, RYR2, RYR3, S100A1, S100B, ATXN1, SCD, SCN5A, SCN8A, CCL2, CCL19, CCL21, CX3CL1, SDC4, CXCL12, SDHB, SDHD, SELP, SGCD, SGCG, SGK1, SGTA, SLC2A1, SLC2A4, SLC3A2, SLC6A2, SLC6A4, SLC6A8, SLC7A1, SLC8A1, SLC9A1, SLC18A3, SLC22A5, SLN, SUMO3, SUMO2, SOAT1, SOD1, SOD2, SOD3, SOX3, SPP1, SPTBN1, SRI, SRF, STAT3, STAT4, STC1, STIM1, SYT1, TAZ, TCF7L2, TERF2, TF, TFAM, TFR2, TFRC, TGFB1, TH, THBS1, THBS2, TIMP1, TIMP2, TIMP3, TIMP4, TJP1, TLR4, TM7SF2, TNF, TNFRSF1A, TNFRSF1B, TNNI3, TNNT2, TOP2B, TP53, TPI1, TPM1, CRISP2, TRAF3, TRPC1, TRPC3, TRPC6, TTN, TTR, TWIST1, TXN, SUMO1, UCP1, UCP2, UCP3, UTRN, VCL, VEGFA, VEGFB, VHL, BEST1, VWF, WRN, XBP1, XDH, YY1, ZBTB17, SLC30A3, MAP3K12, ALMS1, CXCR4, SCG2, MANF, AIMP2, CSRP3, FOSL1, TCL1A, NRIP1, ARID1A, OGT, IKBKG, DENR, PDE5A, DYNLL1, BECN1, DGAT1, CDS2, NR1I2, APLN, MAP3K14, BAZ1B, MAP3K13, SLC33A1, MSC, MAPKAPK2, PPIG, LONP1, ADIPOQ, GRAP2, HAND1, ABCG2, HAND2, GDF15, TBPL1, BAG3, ISG15, ADAMTSL2, NOS1AP, HDAC4, MLEC, TOMM70, TLK1, MFN2, NR2E3, HDAC5, DNM1L, ABCC9, IL18BP, COL4A3BP, LRPPRC, NAMPT, ALYREF, RAMP2, FSTL3, RACK1, PRMT5, FST, SEMA4D, FBLN5, AHSA1, POSTN, CORIN, OGA, NES, LILRB1, PPARGC1A, SUGT1, UTS2, PRDX3, SDS, FSTL1, MAP4K5, NISCH, CHEK2, ECD, ICK, MMRN1, PDS5B, KIF1B, FRMD4B, GPD1L, ARC, PDS5A, SIK3, SIRT3, SIRT1, ZFPM2, NPTXR, NNT, DAPK2, BRD1, RAB3GAP2, RNF19A, POLDIP2, SLC17A5, NOX1, ANKRD1, PPA2, HSPB7, VPS4A, EIF3K, SLC27A6, ACAD9, KLF15, SETD2, TBK1, GRHL1, GNL2, RMC1, KCNIP2, EHD3, DUOX2, NOX4, TNNI3K, RMDN1, GP6, PRKAG2, POLK, ISYNA1, GDE1, GHRL, LUC7L3, RTEL1, MAP3K20, TPCN1, DUOX1, SEMA5B, UGT1A1, RBFOX1, RNF111, TRPM4, TMEM70, SLC52A1, RMDN3, QRSL1, RNLS, CHDH, MAML3, ZCCHC8, SYBU, ENAH, PBK, ZKSCAN7, ERBIN, RETN, CHPT1, NLN, SEMA6A, CFAP97, EPG5, SUGP1, GATAD1, HAMP, CXCL16, RHOU, MYL7, RBM25, ACE2, ELAC2, PRDM16, IFIH1, GIGYF1, GORASP1, PINK1, CDK15, MARCKSL1, WNK1, FTO, ELOVL6, FKRP, GNPTAB, FSD1, TMEM43, NOX5, ZC3H12A, SLC2A10, FSD1L, KAT8, TCHP, HOPX, SPZ1, GTPBP3, CEP19, MYLK2, UCN2, MLIP, NEXN, DNER, ADAMTSL1, MYOCD, MUC16, OPN4, PRRT2, CMTM7, SLC25A26, XIRP2, DNAJC19, PPARGC1B, KLF14, MTPN, LGALS16, HJV, RMDN2, DHRS7C, TUBB, PRSS55, TPCN2, COPD, RBM20, NSMCE2, MALAT1, MIR100, MIR126, MIR134, MIR137, MIR142, MIR150, MIR17, MIR18A, MIR195, MIR199A1, MIR199A2, MIR199B, MIR21, MIR214, MIR216A, MIR22, MIR23A, MIR25, MIR27B, MIR34A, MIR99A, MIR324, MIR328, MIR340, MIR342, TRIM72, MIR423, MIR425, MIR499A, NCF1, MIR539, OPN1MW2, CCR2, RNA18SN5, MIR675, MIR665, MIR208B, BACE1-AS, APELA, CASP12, MIR4491, NPY4R2, PGR-AS1, OPN1MW3, AK6, LOC105379861, LOC10798405 |
