# Supplementary material for: Mechanistic Insights of Qingre Jiedu Recipe Based on Network Pharmacology Approach against Heart Failure
Source: Evid Based Complement Alternat Med. 2022 Jan 31;2022:9024394. doi: 10.1155/2022/9024394 (PMC8820871; doi:10.1155/2022/9024394)
Supplement: Supplementary Materials — Supplementary material related to this article can be found in Supplementary Tables 1, 2, 3, and 4. [file 9024394.f1.zip › 9024394.f1/Supplementary Table 3.docx]

**Supplementary Table 3.Differential Gene.**

| **Differential Gene** |
| --- |
| STAT3, BDNF, GCG, FGF2, CCK, RAC1, CRP, SCD, ADRB1, UCP2, POLR2A, ADRB2, EDN1, IGF2, CTSB, CSF2, MPO, SOAT1, UCP3, SELP, PPARA, BCL2, BAX, PTGS2, PON1, ERBB2, SLC6A2, CASP3, INS, BIRC5, AKR1B1, CASP1, PRKCA, PPARD, MAPK8, DUOX2, IRF1, MMP3, RELA, TNF, PTGS1, SOD1, NOS3, SERPINE1, TP53, SLC22A5, XIAP, COL1A2, PPP3CA, PTEN, PPARG, ACACA, HSF1, TXN, MMP9, MME, CYBB, NCF1, AKT1, HIF1A, CHEK2, NOS2, CASP12, VEGFA, PRKCB, IL1B, HMOX1, CDKN1A, MMP1, SLC2A4, SOD3, FOS, GJA1, IKBKG, IL10, XDH, IL4, IFNG, COL1A1, MMP2, IL6, CXCL2, AHSA1, MAPK1, TRPC3, |
