# Supplementary material for: Mechanistic Insights of Qingre Jiedu Recipe Based on Network Pharmacology Approach against Heart Failure
Source: Evid Based Complement Alternat Med. 2022 Jan 31;2022:9024394. doi: 10.1155/2022/9024394 (PMC8820871; doi:10.1155/2022/9024394)
Supplement: Supplementary Materials — Supplementary material related to this article can be found in Supplementary Tables 1, 2, 3, and 4. [file 9024394.f1.zip › 9024394.f1/Supplementary Table4.docx]

**Supplementary Table 4.hub genes.**

| **hub genes based on the betweenness centrality** |
| --- |
| \| AKT1 \| INS \| IL6 \| PPARG \| TP53 \| TNF \| BDNF \| CASP3 \| EDN1 \| MAPK8 \| VEGFA \| NOS3 \| MAPK1 \| FOS \| PTGS2 \| STAT3 \| SOD1 \| RELA \| PTBN \| EGF2 \| \| --- \| --- \| --- \| --- \| --- \| --- \| --- \| --- \| --- \| --- \| --- \| --- \| --- \| --- \| --- \| --- \| --- \| --- \| --- \| --- \| |

| **hub genes based on the degree** |
| --- |
| \| EDN1 \| IL10 \| MMP9 \| HMOX \| MMP2 \| MPO \| IL4 \| SERPINE1 \| IFNG \| RELA \| HIF1A \| BDNF \| PTBN \| SOD1 \| CRP \| MMP1 \| CSF2 \| ERBB2 \| CASP1 \| NOS2 \| \| --- \| --- \| --- \| --- \| --- \| --- \| --- \| --- \| --- \| --- \| --- \| --- \| --- \| --- \| --- \| --- \| --- \| --- \| --- \| --- \| |
